# Preventing metastatic recurrence in low-risk ER/PR + breast cancer patients—a retrospective clinical study exploring the evolving challenge of persistence with adjuvant endocrine therapy

**DOI:** 10.1007/s10549-022-06849-0

**Published:** 2023-01-02

**Authors:** Elaine P. Kuhn, Jonathan Pirruccello, James T. Boothe, Zhongze Li, Tor D. Tosteson, James E. Stahl, Gary N. Schwartz, Mary D. Chamberlin

**Affiliations:** 1 Department of Internal Medicine, Dartmouth-Hitchcock Medical Center, Lebanon, NH USA; 2 Norris Cotton Cancer Center, Dartmouth-Hitchcock Medical Center, Lebanon, NH USA; 3 Department of Biomedical Data Sciences, Dartmouth-Hitchcock Medical Center, Lebanon, NH USA

**Keywords:** Adherence, Persistence, Compliance, Adjuvant endocrine therapy, Low-risk breast cancer, Oncotype-DX®, Metastatic recurrence, Distant recurrence

## Abstract

**Purpose:**

In the genomic era, more women with low-risk breast cancer will forego chemotherapy and rely on adjuvant endocrine therapy (AET) to prevent metastatic recurrence. However, some of these patients will unfortunately relapse. We sought to understand this outcome. Preliminary work suggested that early discontinuation of AET, also known as non-persistence, may play an important role. A retrospective analysis exploring factors related to our breast cancer patients’ non-persistence with AET was performed.

**Methods:**

Women who underwent Oncotype-DX® testing between 2011 and 2014 with minimum 5 years follow-up were included. ‘Low risk’ was defined as Oncotype score < 26. Outcomes of recurrence and persistence were determined by chart review. Patient, tumor and treatment factors were collected, and persistent versus non-persistent groups compared using multivariable ANOVA and Fisher Chi square exact test.

**Results:**

We identified six cases of distant recurrence among low-risk patients with a median follow-up of 7.7 years. Among them, five of six patients (83%) were non-persistent with AET. The non-persistence rate in our cohort regardless of recurrence was 57/228 (25%). Non-persistent patients reported more severe side effects compared with persistent patients (*p* = 0.002) and were more likely to be offered a switch in endocrine therapy, rather than symptom-relief (*p* = 0.006). In contrast, persistent patients were 10.3 times more likely to have been offered symptom-alleviating medications compared with non-persistent patients (*p* < 0.001). A subset analysis revealed that patients who persisted with therapy had a higher Oncotype-DX® score than patients who discontinued early (*p* = 0.028).

**Conclusion:**

Metastatic recurrence in low-risk breast cancer patients may be primarily due to non-persistence with endocrine therapy. Further work is needed to optimize care for patients who struggle with side effects. To our knowledge, these are the first published data suggesting that Oncotype-DX® score may influence persistence with AET.

## Background

The definition of ‘low-risk’ breast cancer has evolved greatly with our understanding of tumor genetics. In 2004, a 21-gene assay, Oncotype-DX® (ODX) demonstrated significant power to predict 10 years risk of metastatic recurrence in node-negative, estrogen receptor (ER) positive breast cancer patients [1]. ODX uses an algorithm to generate a recurrence score (RS) ranging from 0 to 100. In 2018, Sparano et al. published results of the TAILORx trial and found that patients with RS < 26 had distant recurrence rates less than 5.5% [2], these results updated and refined the definition of ‘low risk’ breast cancer.

Identifying low-risk disease in this context allows for safe de-escalation of treatment and thereby improved quality of life for patients. A key feature of the ODX test is in determining which patients would benefit from chemotherapy. ‘Low risk’ patients have similar outcomes with or without chemotherapy; and in many cases providers will recommend 5 years of adjuvant endocrine therapy alone to prevent metastatic recurrence.

Notably, the ODX 10 years recurrence risk presumes that the patient will complete 5 years of adjuvant endocrine therapy; however, it is well-known that persistence with AET is a formidable challenge. It has been estimated that up to 50% of women discontinue AET early [3]. Further, despite three decades of rich literature on this issue, persistence rates remain largely unchanged [4, 5], as interventions designed to address this problem have been met with limited success [6, 7]. Meanwhile, use of endocrine therapy is expanding to include the neoadjuvant setting [8], and novel oral endocrine therapies show promise for patients with advanced and metastatic disease [9–11]. Persistence with endocrine therapy therefore represents a challenge of increasing relevance in breast cancer care.

The most common reason cited for early discontinuation is side effects. These may include hot flashes (60.7%), arthralgias (74.3%), and cognitive/mood disorders (64.3%), among others [12]. Sexual dysfunction (54.7%) is often underreported and its impact underestimated [13]. A variety of evidence-based treatment options including pharmacologic and non-pharmacologic options are available [14] and included in NCCN guidelines [15].

As an alternative to addressing of side effects directly, patients may “switch” to another endocrine therapy that may be better tolerated. It has been estimated that between 38 and 47% of patients who switch therapy will continue with it [16, 17]. In contrast, others have shown that switching therapy is associated with non-adherence and early discontinuation [3, 18, 19]. Current NCCN guidelines includes switching therapy as a ‘consideration’ for patients with arthralgias [15]. Further evidence is needed to determine when and for whom a switch in therapy should be considered.

While side effects and their management are clearly important, a wealth of literature reveals the issue to be even more complex. Non-persistence has been associated with factors such as patient age [20–22], race and ethnicity [23–25], fertility concerns [26], presence of baseline symptoms [27, 28] and co-morbidities [27] such as mental health disorder [29–31] and substance use disorder [29], receipt of non-specialist care [32], communication with providers [33–36], cost concerns [23, 25, 37–40], and individual beliefs and perceptions about AET [37, 41–43]. Lower tumor stage and negative lymph node status are also associated with non-persistence [19, 44, 45]. There are limited data available on the influence of genetic test results [46], although this may be an area of increasing importance.

What follows is the results of our preliminary work aimed at understanding metastatic recurrence in patients with low-risk breast cancer. Next, we present a retrospective data analysis comparing persistent and non-persistent patients in terms of patient and tumor characteristics, Oncotype-DX score, side effects and their management, in order to better understand non-persistence in our population.

## Methods

A list of patients that had their breast surgery performed at Dartmouth-Hitchcock Medical Center and had Oncotype-DX testing between April 2011 and Dec 2014 was shared by the Department of Pathology at Dartmouth-Hitchcock. Upon IRB approval, a chart review was conducted to extract Oncotype-DX scores from scanned PDF files. Women with a minimum of 5 years follow-up data were included in the study. Low-risk was defined as having localized disease and Oncotype scores < 26 [47]. Recurrence of disease was defined as pathologically confirmed breast cancer at distant/metastatic sites. Development of local recurrence, contralateral breast cancer or a new ipsilateral primary breast cancer was not considered a recurrence event for the purposes of this study. The primary outcome was prevalence of distant recurrence with a minimum follow-up of 5 years in women with low-risk breast cancer.

Based on the initial results, an additional chart review was conducted to determine rates of non-persistence within our cohort. Early discontinuation was determined by office visit notes, and defined as having not completed at least 55 months of therapy with either aromatase inhibitor, tamoxifen, or both. The duration of 55 months was chosen because previous reports have defined similar cutoffs [23, 48]. In our clinical experience, patients who stop a few months prior to 5 years, with greater than 85% persistence, would not be considered a non-persistence event. Additional variables of interest were collected including patient age, location of cancer care, co-morbidities, prior use of hormone replacement therapy and socioeconomic factors. Tumor pathology reports were reviewed for variables including tumor size, grade, lymph node involvement, lymphovascular invasion, positive margins and need for re-excision. Details of the treatment history were recorded including number of months on endocrine therapy, prescription for aromatase inhibitor vs tamoxifen as the initial treatment, presence and severity of side effects, discussion of side effect management versus offering a switch in endocrine therapy as documented in office visit notes, and patient acceptance of these management options. Estimates of 10 years recurrence risk as determined by Oncotype-DX and recurrence score pathological-clinical (RSPC) were collected. Oncotype-DX Recurrence Risk was obtained from the Oncotype-DX report. A free online calculator (genomichealthonline.okta.com) was used to determine RSPC 10 years risk estimates.

Multivariable ANOVA and Fisher Chi square exact test were used to compare these variables in persistent vs non-persistent patients.

## Results

**Fig. 1:**
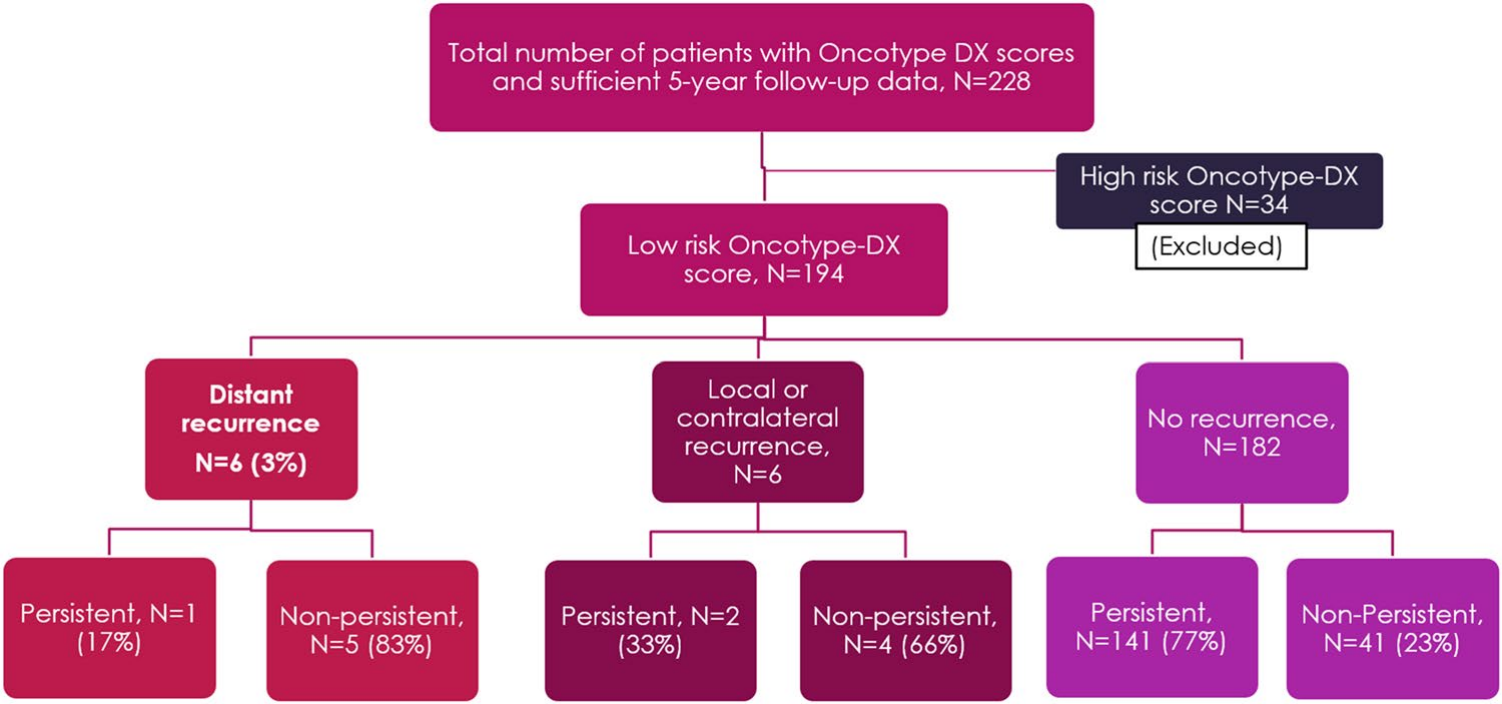
Identification of patients with low-risk breast cancer who experienced distant recurrence within the follow-up period

Of 228 patients who received Oncotype testing between April 2011 and Dec 2014 and had sufficient 5 years follow-up data, 194 (85%) had ‘low risk’ scores. The median follow-up period was 7.7 years. We identified six cases of distant recurrence. Among patients who recurred, 5 of 6 (83%) were non-persistent with endocrine therapy. Among patients who remained disease-free during the follow-up period, 41 of 182 (23%) were non-persistent (Fig. 1).

**Fig. 2:**
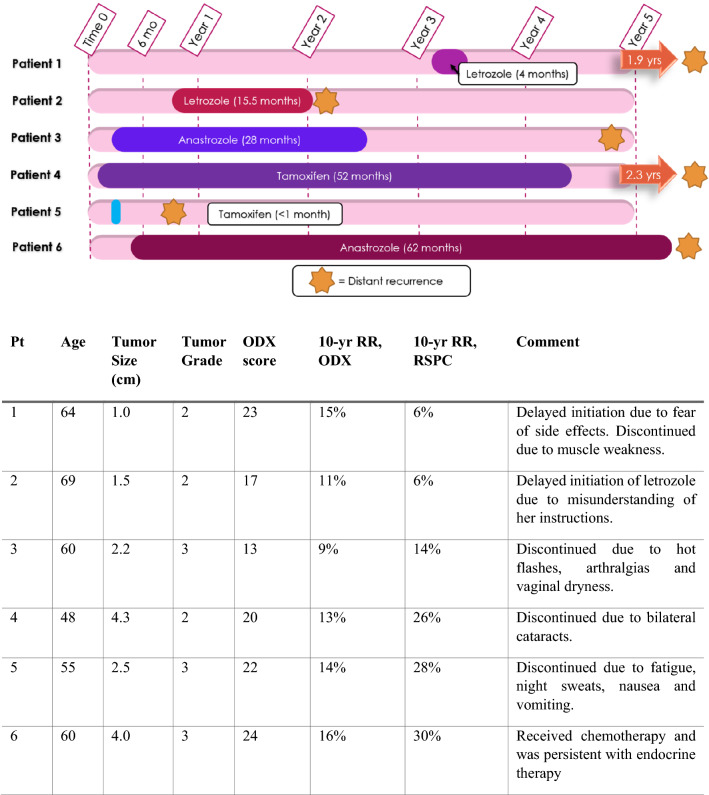
Timeline (drawn to scale) of endocrine therapy duration and time to metastatic recurrence in six patients with low-risk Oncotype. Associated data table includes patient and tumor characteristics, and brief history. Pt patient, ODX oncotype-DX, RR recurrence risk, RSPC recurrence score Pathologic clinical

For each case of distant recurrence, the duration of endocrine therapy and time to recurrence is depicted as a scaled drawing in Fig. 2. Each of these patients had node-negative disease on diagnosis that progressed to metastatic disease of thoracic and/or lumbar spine. In addition, 2 patients (Patients 3 and 6) developed metastatic disease of the liver. Patient age, tumor size, grade, Oncotype-DX score and 10 years recurrence risk is provided in the associated table. Duration of therapy ranged from less than 1 month to 62 months. 4 of 6 patients discontinued therapy early due to side effects, and 2 patients (Patient 1 and 2) had significant delays in treatment initiation (> 6 months). Only one patient (Patient 6) completed the prescribed course of endocrine therapy.

Based on these initial findings, we initiated a deeper chart review to further understand persistence with endocrine therapy in our cohort. For this analysis we included patients with high risk disease.

Of the 228 patients in our cohort, 57 (25%) were non-persistent, defined as having not completed at least 55 months of endocrine therapy. Based on literature review, we compared persistent and non-persistent patients in several variables of interest in order to identify associated factors.

Patient demographics, medical history and socioeconomic history for non-persistent vs persistent patients are shown in Table 1. Groups were similar in age at diagnosis. Nearly all patients (98.6%) in the study were white, non-Hispanic. A significantly greater proportion of patients who completed therapy were noted to have strong or satisfactory social support, compared to those who discontinued early (96% vs. 84%, *p* = 0.002). There was no difference between groups in terms of cost concerns or previous use of hormone replacement therapy. Medical history was not significantly different between groups, and location of care did not differ between groups. A greater proportion of persistent patients participated in RN/NP teaching session prior to surgery compared to non-persistent patients (63% vs. 45%, respectively, *p* = 0.07).

**Table 1:** Patient demographics, medical history, and socioeconomic factors

	Non-persistent with endocrine therapy	Persistent with endocrine therapy	*P*-value
	*n* (column %) or median (standard deviation)		
Age at diagnosis	59 (10.1)	58 (10.6)	0.83
White	57/57 (100%)	168/171 (98%)	
Asian	0/57 (0%)	2/171 (1%)	
Hispanic	0/57 (0%)	1/171 (0.6%)	
Black	0/57 (0%)	0/171 (0%)	
Married at time of diagnosis	37/57 (65%)	131/171 (77%)	**0.08**
Social support noted as strong or satisfactory	47/56 (84%)	164/171 (96%)	**0.002**
Cost was noted as a concern	12/53 (23%)	46/165 (28%)	0.45
Previous use of hormone replacement therapy	14/55 (25%)	39/166 (23%)	0.77
History of hyperlipidemia	16/57 (28%)	60/171 (35%)	0.33
History of COPD	5/57 (9%)	6/171 (4%)	0.11
History of substance use disorder	2/57 (4%)	18/171 (11%)	0.10
History of mental health disorder	24/57 (42%)	66/171 (39%)	0.64
History of joint disease	26/57 (46%)	68/171 (40%)	0.44
Patient received majority of cancer care at DHMC in Lebanon, NH	35/57 (61%)	110/171 (64%)	0.69
Patient participated in RN/NP teaching session prior to surgery (2013–2014 only)	15/33 (45%)	57/90 (63%)	**0.07**

Bold values are highlighting *p* values < 0.05

*DHMC* Dartmouth-Hitchcock medical center

Tumor characteristics are provided in Table 2. There was no significant difference between groups in terms of tumor size or grade, presence of macro- and micro-metastases or isolated tumor cells, lymphovascular involvement, involvement of margins and need for re-excision surgery. Patients in the persistent group had higher Oncotype scores (15.2 vs. 13.7, *p* = 0.086) as well as 10 years risk of recurrence (10.1% vs. 9.2%, *p* = 0.091) compared to non-persistent patients, and this difference was statistically significant for the subset of low-risk patients who reported bothersome side effects (15.9 vs. 13.7, *p* = 0.028). Interestingly, there was no difference between groups in terms of the RSPC 10 years recurrence risk, which incorporates tumor size and grade with Oncotype-DX score. This result is consistent with our findings that tumor size and grade do not appear to influence patient persistence during this study time period.

**Table 2:** Tumor pathology report data and 10 years risk estimates

	Non-persistent with endocrine therapy	Persistent with endocrine therapy	*P*-value
	*n* (column %) or mean (standard deviation)		
Tumor size (cm)	1.92 (1.6)	2.0.05 (1.7)	0.60
Tumor grade	1.84 (0.7)	1.95 (0.7)	0.29
Lymph node involvement by micro or macro metastases	10/56 (18%)	45/171 (26%)	0.20
Lymphovascular invasion	13/57 (23%)	40/169 (24%)	0.89
Margins involved	12/57 (21%)	32/169 (19%)	0.73
Oncotype-DX score (all score ranges)	16.6 (10.4)	18.5 (10.1)	0.22
Oncotype-DX score (low-risk group)	13.7 (6.5)	15.2 (5.2)	0.086
Oncotype-DX score (low risk, + sx)	13.7 (1.0)	15.9 (0.47)	**0.028**
Oncotype 10 years recurrence risk (low-risk group)	9.2% (3.5)	10.1% (2.8)	0.091
Oncotype 10 years recurrence risk (low ris, + sx)	9.4% (3.6)	10.4% (2.4)	0.084
RSPC 10 years recurrence risk (low-risk group)	6.9% (5.9)	7.6% (6.5)	0.475

Bold values are highlighting *p* values < 0.05

*Sx* bothersome symptoms reported, *RSPC* recurrence score pathologic clinical

Groups did not differ in terms of type of surgery (breast conserving vs mastectomy) or receipt of radiation therapy (data not shown).

A significantly greater proportion of patients received a prescription for aromatase inhibitor in the persistent group compared to the non-persistent group (90% vs. 75%, *p* = 0.01). Persistent patients endured endocrine therapy significantly longer prior to switching or discontinuing treatment, with a median duration of 36 months on tamoxifen and 25 months on aromatase inhibitor, compared with 9 months on tamoxifen and 15 months on aromatase inhibitor (AI) (*p* < 0.001 and *p* = 0.026, respectively). The large difference in duration on tamoxifen is likely confounded by the scenario of pre-menopausal women who switch to AI after achieving menopausal status.

**Fig. 3:**
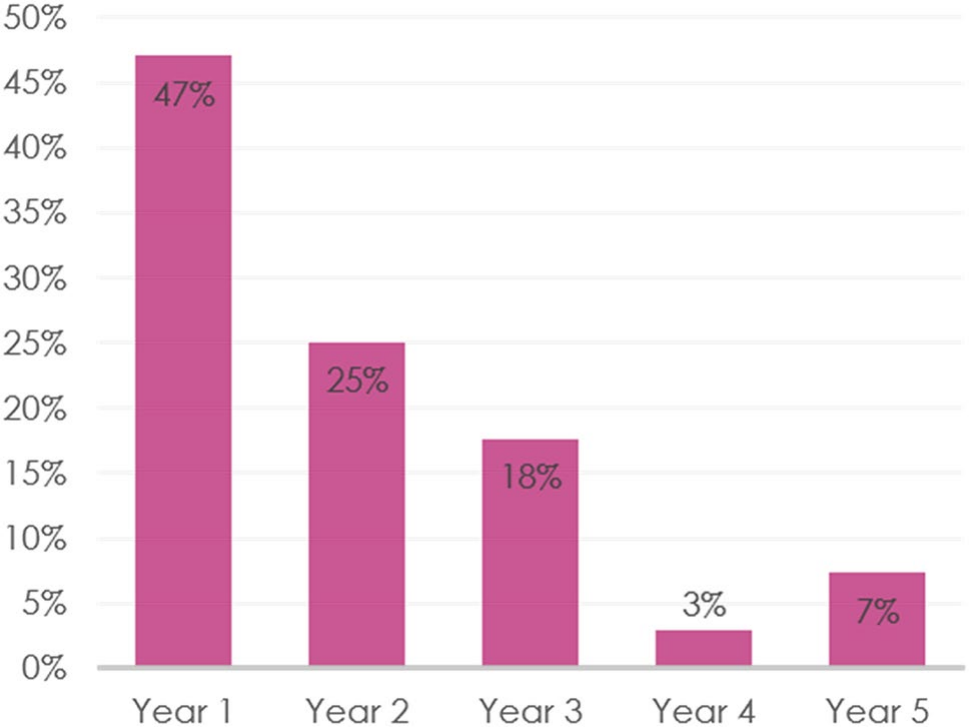
Proportion of non-persistent patients who discontinued therapy during each year of treatment

Nearly half of non-persistent patients discontinued endocrine therapy within the first year of treatment (Fig. 3). The most commonly cited reason for early discontinuation was side effects (74%), followed by cost (13%), unknown (9%) and co-morbidities (4%).

The proportion of patients reporting symptoms attributed to tamoxifen and aromatase inhibitors is included in Table 3. The median number of total side effects reported did not differ between groups. Non-persistent patients were more likely to report severe symptoms for both tamoxifen and aromatase inhibitors compared to persistent patients (78% vs. 43% for tamoxifen, 82% vs. 53% for aromatase inhibitor, *p* = 0.002 for both). Fatigue was the only symptom disproportionately represented among non-persistent patients (33% vs. 12%, *p* = 0.01), for those taking tamoxifen.

**Table 3:** Side effects reported

	Non-persistent with endocrine therapy	Persistent with endocrine therapy	*P*-value
	*n* (column %) or mean (standard deviation)		
Tamoxifen	*N* = 27	*N* = 83	
Hot flash	14 (52%)	55 (66%)	0.18
Insomnia	7 (26%)	20 (24%)	0.85
Mood change	6 (22%)	15 (18%)	0.55
Fatigue	9 (33%)	10 (12%)	**0.01**
Vaginal dryness	1 (4%)	16 (19%)	**0.05**
Other	18 (67%)	32 (39%)	**0.01**
Aromatase inhibitor	N = 37	N = 169	
Arthralgias	25 (68%)	89 (67%)	0.99
Hot flash	17 (46%)	75 (57%)	0.24
Insomnia	4 (11%)	27 (20%)	0.18
Mood change	7 (19%)	14 (11%)	0.18
Fatigue	11 (30%)	26 (20)	0.19
Vaginal dryness	4 (11%)	22 (17%)	0.37
Other	19 (51%)	30 (23%)	**0.001**

Bold values are highlighting *p* values < 0.05

**Fig. 4:**
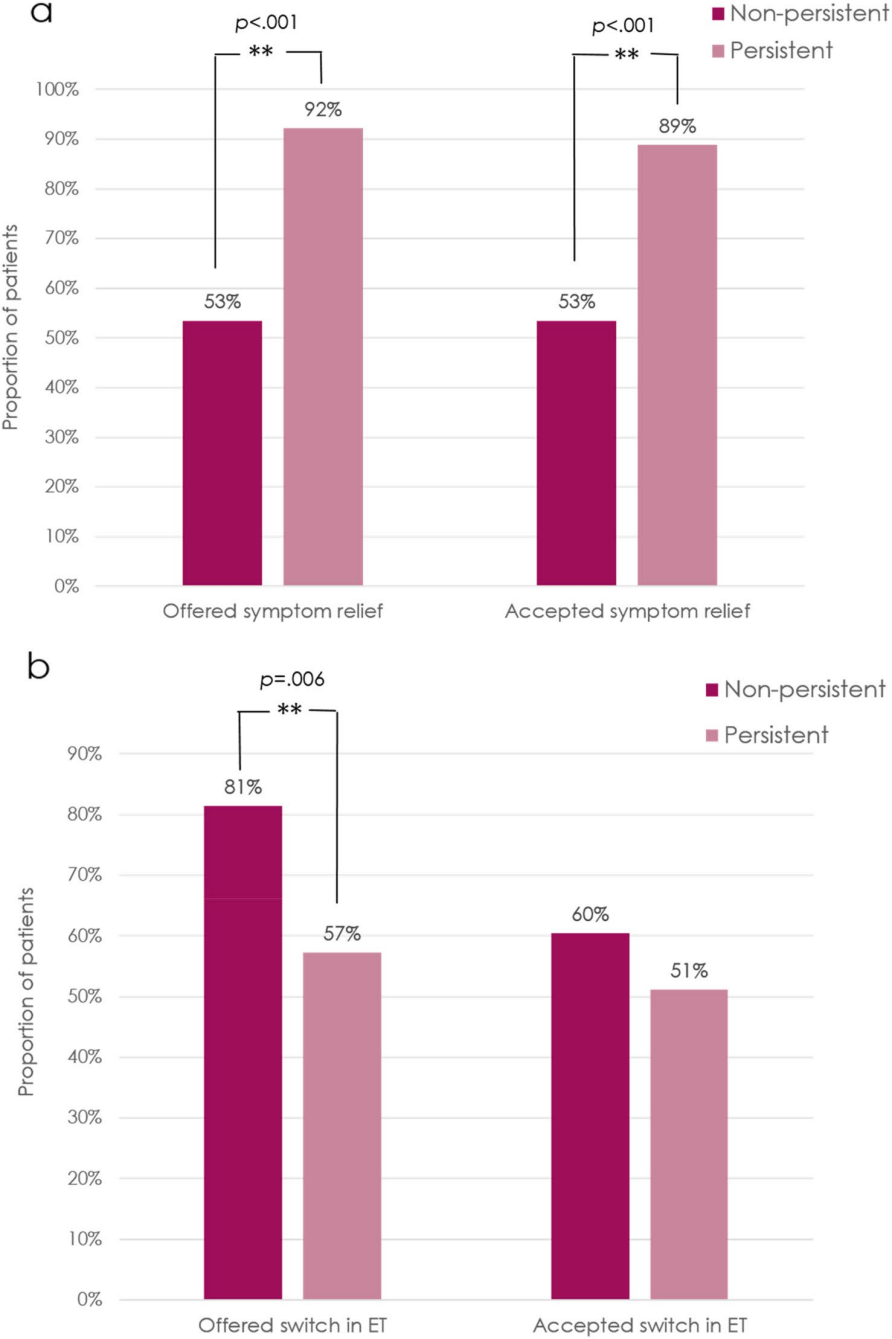
Clinical management of bothersome symptoms attributed to endocrine therapy in persistent versus non-persistent patients. **a** proportion of patients who were offered and accepted symptom relief, **b** proportion of patients who were offered and accepted a switch in endocrine therapy

The clinical management of patients who reported bothersome side effects is shown in Fig. 4. A greater proportion of persistent patients were offered symptom-alleviating medications compared to non-persistent patients (92% vs. 53%, respectively, *p* < 0.001). In contrast, non-persistent patients were more likely to be offered a switch in hormone therapy compared to those who were persistent with treatment (81% vs. 57%, respectively, *p* = 0.006).

In both groups, discussion of side effect management often led to use of medications, with 23/23 of non-persistent patients, and 80/83 of persistent patients accepting medications for side effects. In contrast, persistent patients were more likely to accept a switch in hormone therapy when offered, compared to non-persistent patients (OR 3.18; 95% CI 0.96–10.5), although this difference did not reach significance likely due to the small number of patients in this subset analysis.

## Discussion

We identified six cases of distant metastatic recurrence in a cohort of 194 low-risk Oncotype breast cancer patients with a median follow-up period of 7.7 years, corresponding to an overall recurrence rate of 3%. For persistent patients the recurrence rate was only 0.7% (1/144) and for the non-persistent patients was 8.8% (5/57). This recurrence rate is lower than previously reported by Paik et al., 2004 (> 6.8%) and Sparano et al. 2018 (5.8%). Possible explanations for this including pre-selection of patients with favorable prognosis to receive Oncotype-DX testing, a shorter follow-up period, and increased use of aromatase inhibitors over the last 10 years, which confer better disease-free survival compared with tamoxifen [49].

Among the six cases of recurrence, only one patient completed the recommended course of therapy. Despite her compliance, several factors conferred higher recurrence risk including an Oncotype score of 24, and a high-grade tumor that measured 4.0 cm in size. These clinicopathologic factors are better captured by the Recurrence Score Pathologic Clinical (RSPC) calculator, which would estimate her 10 years risk of recurrence to be 30% (compare to 16% risk estimated by Oncotype-DX). Whether to categorize this patient as ‘low risk’ within this context is an important question [50, 51].

In the remaining five cases of low-risk breast cancer that progressed to metastatic disease, each of those patients either did not initiate or did not persist with endocrine therapy as was recommended. Our findings raised the question of whether most early distant recurrences in women with low-risk Oncotype breast cancer occur in the setting of failure to complete a 5 years course of endocrine therapy. This prompted further work to understand non-persistence in our population.

We found the rate of non-persistence with endocrine therapy in our population to be 25% which is consistent with the United States literature. However, for reasons discussed below and as has been pointed out by other groups, it is likely our data are underestimating the true rate of non-persistence.

Similar to others [31], we found that social support is associated with persistence to endocrine therapy, with 92% of persistent patients reporting strong or satisfactory support, compared with 84% of non-persistent patients (*p* = 0.002). It has been hypothesized that clinical social support can mitigate low personal social support [52], although we did not measure this directly in our study. We found that a higher percentage of persistent patients engaged in RN/NP teaching compared with non-persistent patients, and this difference approached statistical significance (63% vs. 45%, respectively, *p* = 0.07).

We hypothesized that if persistence with medication is motivated in part by an understanding of risk, the 10 years recurrence risk as estimated by Oncotype-DX may be influential. Others have shown the influence of recurrence score on initiation of endocrine therapy [46] however did not find a significant effect on early discontinuation. In contrast, we found that patients who persisted with endocrine therapy despite bothersome side effects had significantly higher Oncotype Scores compared to patients who discontinued early, although the difference was small (15.9 vs. 13.7, *p* = 0.028). To our knowledge, this study is the first to show data suggesting a potential influence of Oncotype-DX score on patient decisions regarding persistence with endocrine therapy.

Previous studies that have shown higher tumor stage and size are associated with persistence with endocrine therapy [21, 53, 54]. In contrast, we found no significant difference between persistent and non-persistent patients in terms of tumor size, grade, lymph node involvement, or need for revision surgery. Lymph node involvement has produced mixed results in the literature [55, 56] and in our study this was the only clinicopathologic factor that trended toward having an effect with 26% of persistent patients having lymph node involvement compared with 18% of non-persistent patients, but this did not reach statistical significance (*p* = 0.20). Additionally, there was no difference between groups in terms of 10 years risk as estimated by the RSPC calculator, which incorporates tumor size and grade (6.9% vs. 7.6%, *p* = 0.475), although it is notable that this calculator was not in routine use clinically during this study period.

There was no difference in the median number of side effects reported between persistent and non-persistent patients; however, non-persistent patients were more likely to report severe symptoms compared to persistent patients (OR 4.11, 95% CI 2.1–7.9, *p* = 0.002). Fatigue was more frequently reported in non-persistent patients compared to persistent patients (33% vs. 12%, *p* = 0.01). To our knowledge, this is the first report implicating fatigue as being significantly associated with non-persistence. Based on our review, the most common side effects associated with non-persistence are gynecologic symptoms [13] and arthralgia [5]. Attributing non-persistence to side effects alone, however, may be an over-simplification [4]. Further, it may be difficult to discern side effects of endocrine therapy from symptoms and perceptions of normal aging. Analysis of the International Breast Cancer Intervention Study (IBIS 1) showed no significant difference between symptom effect size on adherence between patients on tamoxifen vs placebo [57]. Additional support for this concept arose from IBIS 2, where investigators found no difference in adherence rates for women on anastrozole compared to women on placebo (65.7% vs. 65.9%, respectively). In fact, in that study adherence rates were lower in the placebo group than in the treatment group for women with arthralgia [58]. Another group found that, after adjusting for joint pain severity, women with high levels of aging perceptions were at greater risk of non-adherence than women with low levels, suggesting that the association of joint pain with older age compounds the issue of adherence [59].

The IBIS 2 study showed a significant trend toward non-adherence in women on anastrozole who reported gynecologic symptoms. Sexual side effects of endocrine therapy are thought to be underreported by both patients and clinicians [4, 60] and remains an important barrier to persistence with endocrine therapy. Interestingly, we found that patients who complained of vaginal dryness were more likely to persist with tamoxifen vs AI, although this difference did not reach statistical significance (OR 6.2; 95% CI 0.78–49.2; *p* = 0.05). Tamoxifen has a tendency to increase vaginal discharge [61]; it is possible this effect is mitigating vaginal dryness and increasing quality of life for some women, thus enabling persistence with tamoxifen.

Switching endocrine therapy has been associated with poorer persistence [16, 19]. Our study showed that non-persistent patients were 3.8 times more likely to be offered a switch in endocrine therapy upfront rather than first trying symptom-relieving medications (OR 3.8, 95% CI 1.44–10.1; *p* = 0.006). Moreover, compared with the persistent group, a greater proportion of non-persistent patients declined a switch, and this difference approached statistical significance (26% vs. 10%, *p* = 0.05). In contrast, persistent patients were 10 times more likely to have been offered symptom-alleviating medications compared with non-persistent patients (OR 10.3, 95% CI 3.8–27.4, *p* < 0.001). In both groups, nearly all patients who were offered symptom-alleviating medications went on to accept a prescription (100% of non-persistent, and 96% of persistent patients).

A possible explanation for these findings may be the perception that patients experiencing certain side effects are more likely to benefit from medication management versus a switch. A subset analysis (data not shown) revealed that side effect profiles differed between patients who were offered symptom-management versus a switch, specifically, patients with hot flashes were more likely to have been offered side effect management. Patients who switched medications reported a slightly higher proportion of arthralgias and a significantly greater proportion ‘other’ less common side effects, perhaps for which evidence-based management is not readily available. Further, it is possible that when patients are engaged in side effect management with their provider, they may have a more positive communication experience compared with those being offered a switch in therapy. Numerous studies have reported on the association of patient-centered communication and persistence with endocrine therapy [34, 35, 62, 63]. Finally, it is possible that some patients had indeed expressed their disinterest in a trial of symptom-relieving medications, and these discussions simply were not documented in the office notes.

We hypothesize that these data may inform future interventions aimed at improving persistence with endocrine therapy. For example, offering a switch in medication frontline in patients with certain side effects, and encouraging evidence-based side effect management in patients with other symptoms. Additional studies are needed to better understand which patients and side effects are best managed with a switch in endocrine therapy versus side effect management. A clinical trial is under development at our institution to help answer these questions.

There are several limitations to our study. Firstly, this is a retrospective clinical study with data based entirely on chart review. Errors in documentation and patient reporting are surely present to some extent. Records of treatment duration confirmed by pharmacy records were not available. Therefore, it is likely that we have underestimated the rate of non-persistence in our population. Additionally, this was a relatively small, non-diverse sample at a single site in Northern United States. Specifically, 98.6% of the sample population was white and non-Hispanic. Differences in AET adherence by race has been documented, with several reports showing black and Hispanic patients are at higher risk [70]; however, further work is needed to understand this complex issue [64–69]. Interventions to address non-adherence and non-persistence should address racial/ethnic differences and aim to eliminate outcome disparities.

## Conclusion

Distant metastatic recurrence in low-risk breast cancer patients may be primarily due to non-persistence with endocrine therapy. Further work is needed to optimize care for patients who struggle with side effects and to guide the use of switches in endocrine therapy versus side effect management. Further, our data suggests that genomic test results may impact patient decisions to persist with endocrine therapy. Furthering our understanding of this relationship will be important for patient care, now and in the future.

## Data Availability

The datasets generated during and/or analysed during the current study are from the corresponding author on reasonable request.
